# Universal principles of membrane protein assembly, composition and evolution

**DOI:** 10.1371/journal.pone.0221372

**Published:** 2019-08-15

**Authors:** Alan J. Situ, Tobias S. Ulmer

**Affiliations:** 1 Department of Physiology and Neuroscience, Keck School of Medicine, University of Southern California, Los Angeles, CA, United States of America; 2 Department of Biochemistry and Molecular Medicine, Keck School of Medicine, University of Southern California, Los Angeles, CA, United States of America; Nanyang Technological University, SINGAPORE

## Abstract

Structural diversity in α-helical membrane proteins (MP) arises from variations in helix-helix crossings and contacts that may bias amino acid usage. Here, we reveal systematic changes in transmembrane amino acid frequencies (f) as a function of the number of helices (n). For eukarya, breaks in f(n) trends of packing (Ala, Gly and Pro), polar, and hydrophobic residues identify different MP assembly principles for 2≤n≤7, 8≤n≤12 and n≥13. In bacteria, the first f break already occurs after n = 6 in correlation to an earlier n peak in MP size distribution and dominance of packing over polar interactions. In contrast to the later n brackets, the integration levels of helix bundles continuously increased in the first, most populous brackets indicating the formation of single structural units (domains). The larger first bracket of eukarya relates to a balance of polar and packing interactions that enlarges helix-helix combinatorial possibilities (MP diversity). Between the evolutionary old, packing and new, polar residues f anti-correlations extend over all biological taxa, broadly ordering them according to evolutionary history and allowing f estimates for the earliest forms of life. Next to evolutionary history, the amino acid composition of MP is determined by size (n), proteome diversity, and effective amino acid cost.

## Introduction

Membrane proteins (MP), defined as traversing the lipid bilayer at least once, mediate the exchange of metabolites, ions, and information between different cellular compartments. This puts MP in control of key physiological and pathological processes as is evident from the large number of drugs that target MP [1]. Genomes typically contain 20–30% proteins that populate the membrane [2]. Despite their high biological and medical significance, MP are poorly understood especially on a structural level when compared to water-soluble proteins. As proteins traverse the membrane they need to navigate the aqueous cytosol, the chemically complex lipid headgroups, and the hydrophobic lipid hydrocarbon tails [3]. Moreover, arising from lipid diversity and membrane asymmetry [4, 5], this environment is cell-type specific and anisotropic along the membrane normal. Difficulties to reproduce functional lipid environments *in vitro* contribute to the relatively slow progress of MP structural biology and present value in seeking complementary approaches.

MP traverse the lipid bilayer either as α-helices or β-sheets. To fulfill hydrogen bonding requirements when traversing the membrane core, β-sheets must form closed barrel structures [6, 7]. In contrast, intrahelical hydrogen bonding allows single α-helices to traverse the membrane. The larger ensuing freedom in helix-helix compared to sheet-sheet orientations invariably allows a larger structural diversity, which could explain the nearly exclusive use of α-helical MP in all membranes with the exception of bacterial outer membranes. The success of β-barrels in outer membranes may relate to their relatively high aqueous solubility in the unfolded state and robust folding pathway [6]. However, the dominance of α-helical folds prompts us to exclusively focus on α-helical MP.

In the course of evolution, as organisms developed increasingly complex functional needs, the repertoire of MP structures must have expanded to implement these functions. Structural diversity in mainly parallel TM helix bundles invariably relates to helix count and length, the number and location of helix-helix crossings, and the chemical nature of helix-helix interactions. Certain aspects of these properties can be quantified from the amino acid (AA) sequence of MP. The number of TM helices, termed n, and helix length can be predicted computationally [2, 8]. The number and location of helix-helix crossings often correlates with Pro-mediated helix kinks [9–11]. Helix-helix interactions regularly utilize recurring motifs centering, for example, on glycine packing or polar interactions [9, 12–14]. Such properties appear difficult to interpret for individual MP. We therefore analyzed the frequencies of all TM AA for entire biological taxa as a function of n, which allowed not only insight into universal assembly principles of MP but also into the drivers of MP diversity, AA composition, and evolution.

## Results and discussion

### The size distribution of MP peaks earlier in bacteria than eukarya

**Fig 1:**
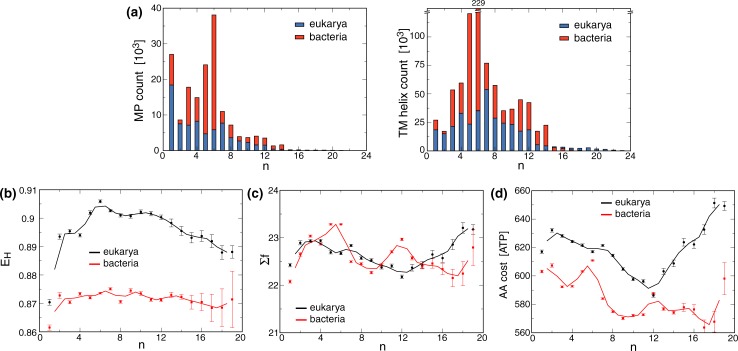
Select membrane protein parameter as a function of n in eukarya and bacteria. (a) Size distribution of MP. The protein count and the count of the total number of TM helices are depicted as a function of n for n≤23. (b-d) AA diversity quantified by the Shannon equitability index (E_H_), TM helix length (Σf, i.e., sum of f over all AA), and AA cost per TM helix in terms of high-energy phosphate bonds (ATP) are shown as a function of n for n≤19. Solid lines depict running averages calculated with a window size of two. The n range was limited because of the scarcity of MP for very large n.

To achieve an even coverage of TM sequence space at a resolution that is expected to include most of the currently existing MP structures while still allowing variability at non-conserved sequence positions, we studied the representative protein clusters compiled in the UniRef50 database. First, we provide an overview of MP size distribution for eukarya and bacteria. For bacteria, the distribution peaks at n = 6, which is earlier than for eukarya for which a large number of MP entries was obtained until n = 7 (Fig 1A). When excluding MP with n = 1, 73.8% of sequences fall within 2≤n≤6 in bacteria, whereas 75.0% of eukaryotic sequences reside within 2≤n≤7 and only 61.0% within 2≤n≤6. Eukarya further employ a higher percentage of large MP (n≥8) than bacteria. MP numbers decrease substantially for n>12 with eukarya again exhibiting larger counts than bacteria. Thus, eukarya may operate with more complex MP than bacteria.

Bacteria have larger absolute number of sequences in the UniRef50 database (Fig 1A), however, this is likely a reflection of a wider coverage of sequence space rather than a larger number of unique MP folds. Moreover, a wider coverage does not mean that AA diversity is higher in bacteria than eukarya. AA diversity, evaluated by the Shannon equitability index (E_H_) is in fact lower in bacteria than in eukarya for all n (Fig 1B), further suggesting that eukaryotic MP are chemically more complex than their prokaryotic relatives. The wide bacterial coverage of sequence space with a smaller set of preferred AA can arise from extensive AA permutations at non-conserved sites.

### Preferences in AA position along the membrane normal indicate recurring MP architectures and the ability of AA to partake in helix-helix interactions

**Fig 2:**
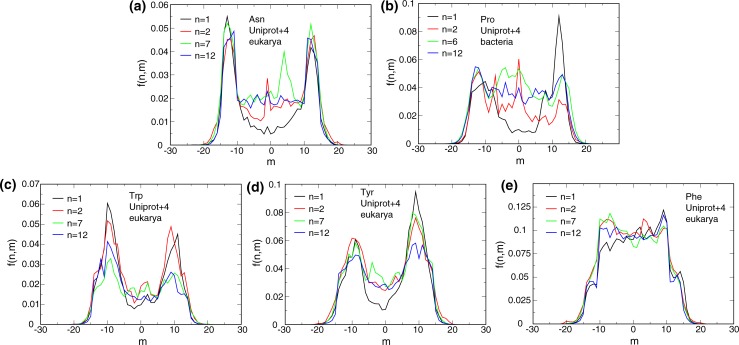
Amino acid frequencies relative to the center of the membrane. f(n,m) profiles of Asn, Trp, Tyr and Phe for eukarya and Pro for bacteria for n = 1, 2, 7 and 12. m denotes the residue number relative to the predicted TM helix center. Negative and positive m values indicate orientations toward the N- and C-terminus, respectively, and do not relate to extra- and intracellular orientations. Fixed UniRef50/Uniprot TM helix borders were used and extended by four residues on either helix side.

Before examining AA frequencies, termed f, as a function of n, we investigated select f(n) profiles that were expanded to individual residue positions along the membrane normal, termed m, with the TM helix center taken as m = 0. Studies of immersion depth preferences of AA provide valuable insight into MP architecture [15–18]. The f(1,m) profile of Gly for example discerns the common GxxxG helix-helix association motif [13] within the N-terminal half of TM helices of eukarya (m = -1, -5; S1A Fig). In Asn of eukarya, f(n,m) profiles exhibit distinct peaks for f(2,-1) and f(7,4) wheras for Pro in bacteria m = -7, 0 and m = -4, 0 stand out for n = 2 and n = 7, respectively (Fig 2A and 2B). As another example, Tyr of eukarya shows a conspicuous peak at f(7,-4) (Fig 2D). The recurring use of certain m positions in helix-helix interactions and their specific variation with n may be useful for understanding and modeling the underlying MP architectures.

**Fig 3:**
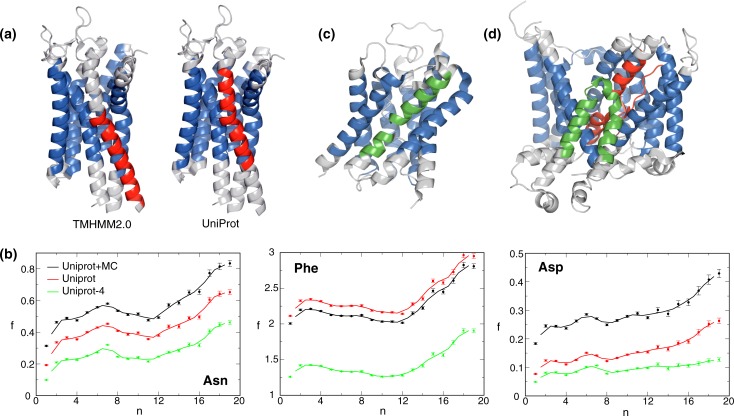
Illustration of uncertainties and ambiguities in transmembrane helix definitions. **(**a**)** Comparison of TM helix predictions between UniRef50/UniProt and TMHMM 2.0 for the human free fatty acid receptor 1 (FFAR1; PDB entry 4phu). Helix 5 is shown in *red* and the other TM helices in *blue*. UniProt and TMHMM predictions of helix 5 encompass residues 179–200 and 188–210, respectively. (b) For the depicted AA, f(n) was calculated using UniProt TM borders plus Monte Carlo simulations (*black*), UniProt TM borders (*red*), and UniProt TM borders shrunk by four residues on each side (*green*), respectively. Solid lines show running averages calculated with a window size of two. The n range was limited to ≤19 because of the scarcity of MP for large n (Fig 1A). (c) Structure of Aquaporin from *Methanothermobacter marburgensis* (AqpM; PDB entry 2f2b). The intramembranous helices are colored in *green* and TM helices in *blue*. AqpM is annotated as n = 6 but its structure reveals that two short intramembrane helices combine to form the functional equivalent of a seventh TM helix. (d) Structure of the C-terminal membrane domain of the human erythrocyte anion exchanger 1 (Band 3; PDB entry 4yzf). The intramembranous helices are colored in *green*, discontinuous TM helices in *red* and regular TM helices in *blue*. In addition to its 12 TM helices and UniRef50/UniProt n = 12 annotation, Band 3 contains two relatively long intramembraneous helices and has been considered to have the equivalent of 14 TM helices [24]. Moreover, Band 3 exhibits two unusual, discontinuous TM helices, where helical conformation breaks down near the center of the membrane, which is then traversed in extended conformations.

The f(n,m) profiles of a number of residues identified pronounced f differences between peripheral and center positions. For example, the highest f values of Asn were found near the membrane border irrespective of n in confirmation of an important role in TM helix capping (Fig 2A). In addition, the well-established preference of Trp and Tyr for lipid headgroups [19–21] was reflected by high and low frequencies for peripheral and center m, respectively, and contrast the even f(m) profile of Phe (Fig 2C–2E). Nonetheless, with increasing n, Trp and Tyr behaved differently. For Trp, peripheral f dropped significantly and central f rose modestly. In contrast, Tyr maintained high peripheral f and substantially increased central f. For assembled MP, helix-lipids contacts decrease with increasing n (for example, see Fig 3A, 3C and 3D). Any anchors that merely stabilized individual helices in the course of folding must, upon assembly, invariably partake in helix-helix interactions. Accordingly, we ascribe the enduring usefulness of Tyr at high n to an ability to partake in helix-helix interactions, whereas Trp appears less capable in this respect. Although Trp is a stronger anchor than Tyr [20, 21], a more facile integration of Tyr into helix bundles may contribute to its more frequent use than Trp.

### Uncertainties in TM helix predictions still permit the reliable assessment of relative f(n) changes

The TM border predictions contained in the UniRef50/UniProt records employed herein are generally based on sequence-based computational predictions [2, 8]. We have assessed the accuracy of such predictions by comparing them to structure-based predictions (Fig 3A) [22, 23] and evaluated the impact of resulting uncertainties on f(n). An uncertainty of sequence-based TM border predictions of 3.8 residues was estimated (see Methods) and Monte Carlo simulations were performed to assess the impact of this uncertainty on f(n). For residues that tend to be more abundant outside than inside of the membrane, f values increased and *vice versa* (Fig 3B and S2 Fig). In case of charged residues, this trend involved changes by up to a factor of two. Nonetheless, even for these extreme changes in absolute f values, relative f(n) changes were little affected for n≥2. As an alternative assessment, TM helices were uniformly shrunk by four residues from UniRef50/UniProt borders in order to exclude most residues outside of the membrane. With lesser residues in the TM helices, absolute f values decreased for all residues (Fig 3B and S2 Fig). However, consistent with mere offsets, changes in relative f(n) were again small. The largest change in relative f(n) was seen for Trp, which is explained by the overrepresentation of this residue in the lipid headgroup region (Fig 2C). Thus, f(n) trends can be reliably computed and the Monte Carlo dataset, which includes the lipid headgroup region, was chosen for further analysis.

**Fig 4:**
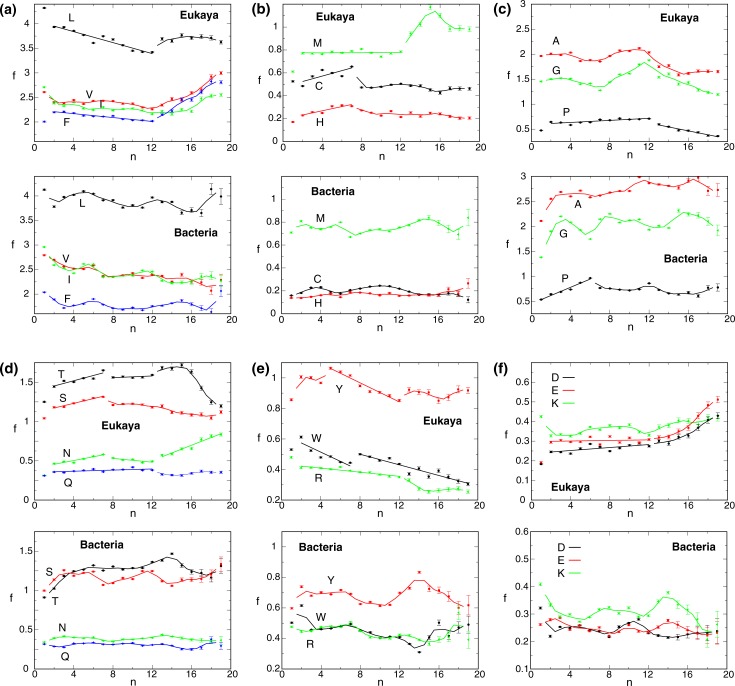
Transmembrane amino acid frequencies as a function of n for eukarya and bacteria. To illustrate f(n) trends, solid lines depict either linear fits or running averages calculated with a window size of two. The n range was limited to ≤19 because of the scarcity of MP for large n (Fig 1A). Additional taxon-specific plots are provided in S3–S7 Figs.

Next to f uncertainties from TM borders, such uncertainties can also arise from ambiguities in n predictions. Marginally hydrophobic helices may evade computational detection, unduly lowering n. Similarly, any MP oligomerization is not taken into account and would effectively increase n. Finally, the complexity of MP structures themselves can give rise to n uncertainties (Fig 3C and 3D). Uncertainties in n misclassify the contribution of affected MP to f(n), which would make transitions between adjacent f(n) values less distinct. However, clear f(n) trends are discernible at the current level of n accuracy (Fig 4).

### f(n) trends in eukarya reveal functional AA groupings and indentify three n regions of distinct MP assembly principles

**Fig 5:**
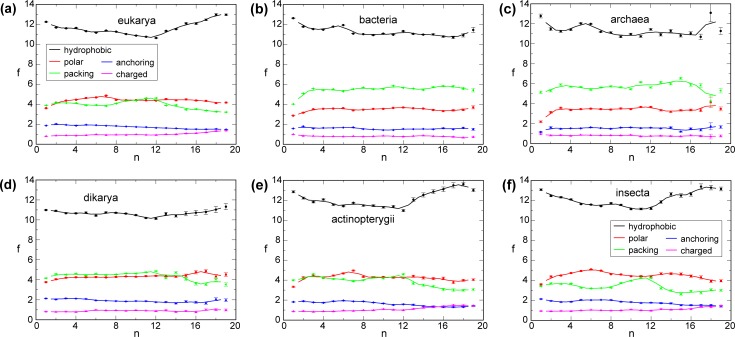
Transmembrane amino acid frequencies as a function of n for functional amino acid groupings. f(n) of hydrophobic (Leu, Met, Ile, Val and Phe), polar (Cys, His, Asn, Gln, Ser and Thr), packing (Ala, Gly and Pro), anchoring (Trp, Tyr and Arg), and charged (Asp, Glu and Lys) residues were combined for each of the depicted biological taxon. Solid lines show running f(n) averages calculated with a window size of two. The n range was limited to ≤19 because of the scarcity of MP for large n (Fig 1A).

For Leu, Met, Ile, Val and Phe, f(n) decreases almost linearly or remains flat up up to n = 12 after which this trend breaks down and a substantial increase in hydrophobic residues is observed (Fig 4A and 4B). Apparently, the previously prevailing MP assembly principles changed to accomplish the folding of very large MP. In the 1≤n≤12 range, f_hydrophobic_ = f_Leu_+f_Met_+f_Ile_+f_Val_+f_Phe_ decreased by 13.2% (Fig 5A), which does not correspond to the large reduction in lipid-exposed surface area in the folded state of MP (Fig 3C and 3D). A lower f_hydrophobic_ limit may arise from the requirement to embed most individual TM helices in the membrane in the unfolded MP state [25]. Complementary to the decrease of f_hydrophobic_, the frequency of polar residues increased with n albeit only initially. f values of Cys, His, Thr, Ser and Asn increased linearly up to n = 7 and, thereafter, mainly decreased (Fig 4B and 4C). Only Gln showed a consistent but less steep f increase up to n = 12. For n≥13, f trends often changed again and initial f values were even superseded for Thr and Asn. Next to the f_hydrophobic_ division between 2≤n≤12 and n≥13, the breaks in f_polar_ trends identify further differences in prevalent MP stabilization between 2≤n≤7 and 8≤n≤12 (Fig 5A).

Gly and Pro have prominent roles in the packing of TM helices [9–11, 13, 14]. As such, f_Gly_ and f_Pro_ may increase with n. Except for a slight weakness at n = 3, this expectation was fulfilled by Pro up to n = 12 (Fig 4C). For Gly, an initial f uptrend reversed at n = 3 to reach a local minimum at n = 7 but substantially increased again up to n = 12. It appears that Gly, as f_polar_ increased for 2≤n≤7, was not in high demand but became more useful as f_polar_ diminished for 8≤n≤12. For n≥13, both f_Gly_ and f_Pro_ trends changed and became less frequent. Above, we did not classify Ala as a hydrophobic residue [26]. Because of its small methyl side chain, Ala may constitute a passive, hydrophobic or a packing-mediating, structural residue like Gly. The f(n) pattern of Ala followed Gly and not hydrophobic residues (Fig 4A and 4C), suggesting that it still can encode helix-helix packing interactions. Nonetheless, its shallower f(n) pattern compared to Gly is noted, which indicates less favorable packing interactions compared to Gly. We thus identify Ala as a packing residue and reveal a complementary and largely balanced nature of f_packing_ and f_polar_ in eukaryotic MP (Fig 5A and 5D–5F).

Similar to the lower limit of f_hydrophobic_, any decrease of f_Trp_ and f_Tyr_ may be limited by a need to anchor TM helices in the unfolded MP state. For Trp, this expectation was not fulfilled; f_Trp_ decreases significantly for 2≤n≤7 due to mostly lower frequencies in headgroup regions (Fig 2C), and this trend continues for n≥8 but less steeply. In contrast, f_Tyr_ remains near maximal values even in headgroup regions up to n = 7 (Figs 2D and 4E). Nonetheless, beginning at n = 5 f_Tyr_ also decreases approximately linearly up to n = 12, after which f_Tyr_ recovers somewhat and remains nearly constant. The divergent f_Trp_ and f_Tyr_ behaviors reinforce our view that Tyr is easier to integrate into TM helix bundles than Trp.

Charged residues also populate lipid headgroup regions where anionic residues contribute to MP anchoring [27, 28]. Asp, Glu and Lys exhibit similar f(n) behaviors; a tendency for a slow f increase in the 2≤n≤12 region that becomes more pronounced for n≥13 (Fig 4F). In contrast, Arg shows a decreasing f trend that becomes pronounced for n≥13 (Fig 4E). This trend is more similar to the amphiphilic Trp and Tyr anchors than charged Asp, Glu, and Lys. In accordance with the delocalized charge of its guanidino group, Arg may have higher amphiphilic capabilities than these residues, which may be useful in the context of some lipid interactions [29, 30]. We further note that Arg has the highest f values among charged residues for small n. Based on the similarity of Arg with Trp and Tyr, we included it in f_packing_ and employed f_charged_ solely for Asp, Glu and Lys.

In sum, the f(n) profiles of eukaryotic MP suggest a functional AA grouping into hydrophobic (Leu, Met, Ile, Val and Phe), polar (Cys, His, Asn, Gln, Ser and Thr), packing (Ala, Gly and Pro), anchoring (Trp, Tyr and Arg), and charged (Asp, Glu and Lys) residues. The corresponding f(n) trends indicate the existence of three n regions with distinct MP assembly principles: 2≤n≤7, 8≤n≤12 and n≥13.

### Bacterial MP rely on packing over polar interactions

In bacteria, the clearest indication of an increasingly integrated TM helix bundles is provided by f_Pro_ for 1≤n≤6 (Fig 4C). This frequency rose from approximately 0.5 to 1 before dropping and staying near 0.75. For n = 1, it experienced large contributions originating in lipid headgroups (Fig 2B) indicative of helix capping and linker modulation [31]. For higher n, central positions became abundant, confirming Pro to aid the tight packing of TM helix bundles [10, 11]. Like in eukarya, f_Gly_ and f_Ala_ appears cyclical and, after an initial rise, forms a local minimum for n = 6. In accordance with the drop in bacterial MP structures after n = 6 (Fig 1A), it appears that the prevailing MP assembly principles already changed at this earlier n value. A second distinction from eukaryotic MP is the slow rise of f_polar_ (Fig 5A and 5B). For example, for Asn, Ser, and Cys, f rises only up to n = 3 and remains relatively steady thereafter (Fig 4B and 4D). For Ser, Thr, Trp, and Tyr there is again a change in f trends at n = 12, which warrants a distinction of MP architecture between 7≤n≤12 and n≥13, although this transition is less pronounced than for eukaryotic MP. Further differences to eukarya were present in anchoring profiles; f_Trp_, f_Tyr_ and f_Arg_ increased up to n = 7 before declining and increasing again for large n (Fig 4E). Moreover, f_charged_ gravitated to lower values with increasing n in contrast to the eukaryotic trend (Fig 4F). Next to differences in relative f trends, absolute f values also differed between eu- and prokarya. The absolute f_polar_ and f_anchoring_ values of bacteria remained below the eukaryotic values in apparent correspondence to the bacterial reliance on f_packing_ (Fig 5A and 5B). Thus, there are clear differences in relative f trajectories between eu- and prokaryotic MP that relate to different size maxima (n = 6 vs 7) and place different weights on polar and packing interactions in assembling TM bundles.

### Domain formation and definition in MP

**Fig 6:**
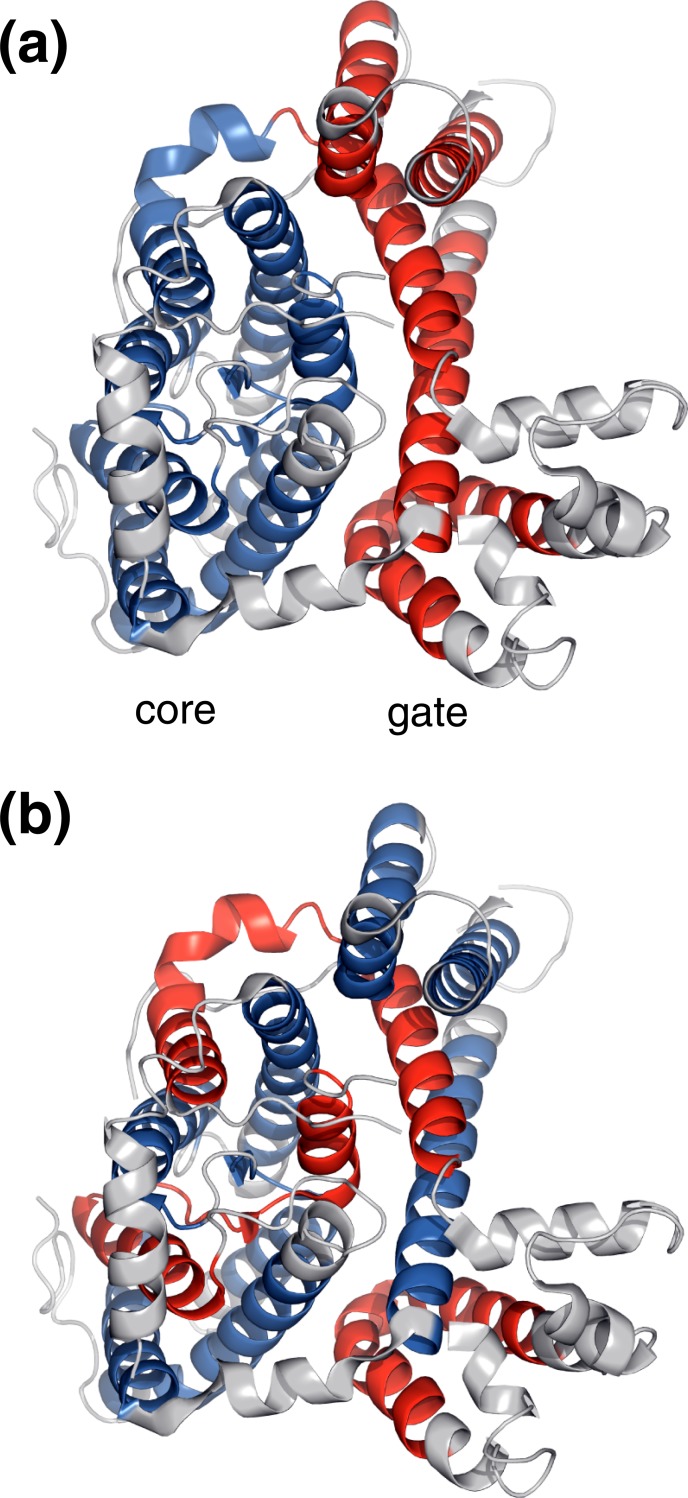
Domain definition in the C-terminal membrane domain of the human erythrocyte anion exchanger 1 (Band3). Band 3 carries out chloride/bicarbonate anion exchange across the plasma membrane of erythocytes [24]. (a) A core (helices 1, 2, 3, 4, 8, 9, 10, and 11) and a gate (helices 5, 6, 7, 12, 13, and 14) domain were differentiated in Band3 [24]. (b) Nonetheless, enzymatic activity of Band3 can be reconstituted by fragments encompassing helices 1–8 and 9–14, shown in *blue* and *red*, respectively [34].

For water-soluble proteins, the concept of protein domains acting as independently folded units has been highly successful [32, 33]. In the context of MP, the term domain is often used to describe a subset of TM helices based on a perceived functional context or evolutionary lineage, whereas its folding independence has usually not been verified. For example, the structure-function relationship of the C-terminal membrane domain of the human erythrocyte anion exchanger 1 (Band3) suggests a core and a gate domain (Fig 6A) [24]. However, it is unclear if both domains, which share an extensive interface, would fold independently of each other. Outside of the native cellular and membrane environment of Band3, an inability to fold would be difficult to interpret. Moreover, Band3 fragments encompassing helices 1–8 and 9–14, which could not fold independently because of voids from missing TM helices (Fig 6B), reconstituted enzymatic activity nonetheless [34]. In MP, it may frequently be ambiguous to differentiate between protein splits and domains. The already folded nature of unassembled, individual TM helices in the membrane may be a source of this ambiguity (and makes individual helices domains, too). Here, we posit that f(n) trends relate more directly to the ability of MP to form *bona fide* domains.

To create structurally diversity in MP, helix-helix crossings and contacts must vary to form helices that are not parallel relative to each other but still packed without voids [35]. f_Pro_ may be most indicative of the level of such diversity as proline-induced helix kinks are central to tightly pack TM helices [10, 11]. For bacterial MP, f_Pro_ increases for 2≤n≤6 before a break to lower f_Pro_ occurs (Fig 4C). Similarly, at n = 6 f trends of Ser and Thr break and level off, respectively (Fig 4D). In addition, the f_Gly_ trend changes most conspicuously at n = 6 (Fig 4C). Thus, helix bundles with n≥7 appear less integrated than 2≤n≤6 proteins, which suggests the formation of a single domain in the latter and raises the possibility of more than one domain in the former range. With smaller absolute f_packing_ values than prokaryotic MP, eukarya appear to place more weight on increasing f_polar_ values to create structural diversity (Fig 5A and 5B). The polar trend conspicuously broke at n = 7 and mainly diminished (Fig 4), suggesting that helix bundles behaved as an integrated unit in the 2≤n≤7 range. For 8≤n≤12, the diminishing f_polar_ was increasingly compensated by f_packing_ in the background of a continued decline of f_hydrophobic_ (Fig 5A and 5F). As such, the TM bundle may or may not be organized as a single unit in this n range. However, the continued decline of f_hydrophobic_ suggests that any domain-domain interface is not lipid exposed and, thus, difficult to dissociate. Band3 could fall in this category (Fig 6A). Moreover we make a contextual reference to the difficulty of separating apparent domains in voltage-gated channels [36, 37]. The f_hydrophobic_ trend breaks down at n = 12 and increases thereafter to even supersede the values for small n. Likewise, f_packing_ breaks down at n = 12 and Asn, Thr, and Tyr increase again. This could be reconciled by two or more domains that do not share a deeply integrated interface. When overlaying the n ranges of possible domain formation on MP counts (Fig 1A), it appears that only relatively few MP form multiple domains. In conclusion, rather than ascertaining the folding independence criterion, the term domain in MP is perhaps best based on functional, structural, and evolutionary relationships.

### AA diversity, the number of TM helices and TM helix length drive MP diversity

Not only MP counts seem to correlate with the breaks in f trends but also additional parameter. TM helix lengths, i.e., Σf, exhibited local n = 5–6 and 12 maxima for bacteria (Fig 1C). In contrast to eukarya, bacteria may be unable to increase AA diversity, i.e., E_H_, with n (Fig 1B) and are left to heighten MP diversity by increasing TM length (Fig 1C). A low AA diversity in bacteria allows for a low metabolic cost of synthesizing the component AA of TM helices (Fig 1D). Nonetheless, the energy investment into lengthening TM helices at n = 5–6 and 12 is apparent. Eukaryotic MP displayed a trend towards shorter TM helices in the 4≥n≥12 range that is reversed for n≥13 (Fig 1D). Evidently, eukarya achieve higher E_H_ levels than bacteria by using more expensive AA that seemed to eliminate the need for longer TM helices for n≤12 (Fig 1D). For both eukarya and bacteria, we note that TM helix costs decrease significantly after MP counts drop off after n = 7 and 6, respectively (Fig 1D). At n = 12, biosynthetic costs between eukaryotic and prokaryotic MP even matched. Eukaryotic MP also increase E_H_ up to n = 6 after which a slow decrease was observed (Fig 1B). Thus, MP complexity is a function of n, E_H_ and Σf. Moreover, we interpret the high AA cost and high Σf in bacteria for 1≤n≤6 as well as the even higher AA costs and high E_H_ in eukarya for 1≤n≤7 as efforts to maximize MP diversity in these single-domain n brackets. In contrast for larger MP, AA cost, Σf, and E_H_ mostly decline up to n = 12 (Fig 1B and 1D) further indicating that the increase of n is sufficient to achieve MP diversity. At n = 12 bacteria form an exception to this statement by adopting unexpectedly high Σf in apparent requirement of heightened diversity. In eukarya, even larger MP (n≥13) finally rely on high Σf instead of high E_H_ at nonetheless relatively high overall AA costs.

### AA frequencies in MP are taxa specific and adopt universal ratios

**Fig 7:**
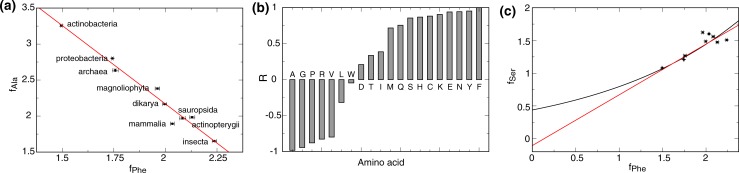
Taxa-specific correlations between pairwise amino acid frequencies (universal ratios). The frequencies of most AA are correlated with each other and with taxonomic association. (a) In the 2≤n≤7 range, f_Phe_ and f_Ala_ of the nine taxonomic groups shown were averaged and correlated using linear equations (S1 Table). (b) The magnitudes of the correlation coefficient, termed R, of analogous linear f_X_-f_Phe_ fits are shown (S8 Fig). (c) While it is difficult to ascertain f_X_-f_Phe_ non-linearity for f_Phe_≥1.5, differences between linear and exponential fits become pronounced for f_Phe_<1.5 as illustrated (see also S8 and S9 Figs and S1 and S2 Tables).

The apparent reliance of bacteria on packing interactions in combination with a scarcity of polar contacts is shared by archaea (Fig 5B and 5C). This may mean that prokaryotes cannot incorporate marginally hydrophobic helices to the same extent as eukarya and that even a modest increase of polar interactions can avoid the need for extensive packing interactions. Moreover, the later peak of eukaryotic as compared to bacterial MP sizes (n = 7 vs 6; Fig 1A) may signal that, by balancing f_packing_ and f_polar_, larger and more complex MP can be formed within a single domain. Notwithstanding these statements, we also found f_packing_-f_polar_ differences between eukaryotic taxa (Fig 5D and 5F). In fact, we found that the frequencies of most AA were correlated and taxa-specific. A specific f_Phe_ for instance not only restricts the possible taxa associated with an MP but also puts tight limits on the frequencies of most other residues. For example, f_Ala_-f_Phe_ are anti-correlated and fit a linear relationship (Fig 7A). It thus appears that the large f discrepancies between bacteria and eukarya are the result of a gradual evolutionary process.

To describe the correlation between AA frequencies systematically, we have ordered AA along the magnitudes of their correlation coefficient, termed R, from linear fits with a reference AA in the 2≤n≤7 range. We chose Phe because it gave the largest sum of R^2^ of all AA. The comparison of R shows that, with increasing f_Phe_, the frequencies of Ala, Gly, Pro, Arg, Val and Leu decrease, Trp is little changed, whereas Asp, Thr, Ile, Met, Gln, Ser, His, Cys, Lys, Glu, Asn and Tyr increase (Fig 7B). This pattern bears some resemblances to the order in which AA were presumably added to the genetic code [38–40]. It is assumed that AA that have been absent from the prebiotic environment were added later, once enzymes for their biosynthesis evolved [41]. Phe is a "new" AA and its rise led to the decrease of "old" AA. In the evolution of mostly soluble proteins, f_new_-f_old_ anti-correlations have been documented and referred to as the universal trend [39, 42, 43]. In analogy, we refer to the detected f_X_-f_Phe_ ratios in MP as universal ratios (S1 and S2 Tables).

### Balancing packing and polar interactions likely benefitted the evolution of complex organisms

**Fig 8:**
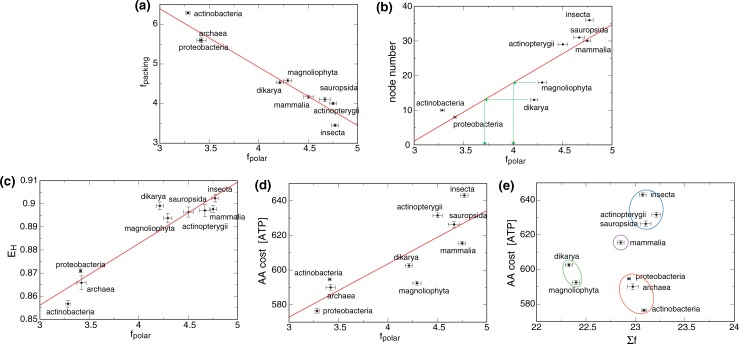
Correlation of membrane protein and evolutionary parameters with taxa-specific polar AA frequencies. To gain insight into MP structural parameter, AA composition, and evolution, f_polar_ was correlated with f_packing_, node number, AA diversity (E_H_), and AA cost for the nine taxonomic groups shown. Additionally, AA cost was correlated with TM helix length (Σf). If applicable, the examined parameters were averaged in the 2≤n≤7 range. Node numbers of taxa refer to the node numbers (number of speciation events) of corresponding model organisms shown in Table 1 and were taken from ref. [44]. AA cost refers to the AA cost per TM helix in terms of high-energy phosphate bonds (ATP) [45]. AA diversity is quantified by the Shannon equitability index (E_H_). TM helix length is the sum of f over all TM AA.

With f_packing_ made up of the old AA Ala, Gly and Pro and f_polar_ comprised of mostly new AA, it is unclear whether f_packing_-f_polar_ balancing is just a byproduct of nearly net neutral evolution or, in relation to functional advantages, drivers of the observed evolution. The phenotypical complexity of organisms in each of the examined taxa broadly increases with f_polar_; simple unicellular prokaryotes gave rise to more complex unicellular eukaryotes, which led to plants and metazoa that formed the most complex taxa (Fig 8A). Consequently, the f_polar_ coordinate relates to evolutionary history. Previously, AA frequencies were proposed to relate to the number of accepted mutations with the number of speciation events (node number) that led to an organism as indicator of this process [44]. For the node number of model organisms, we observed a correlation with taxa-specific f_polar_ except for dikarya and magnoliophyta that adopted higher f_polar_ values than expected (Fig 8B). These outliers indicate that a rise of f_polar_ at the expense of f_packing_ was advantageous; the elevated f_polar_ achieved AA diversities for dikarya and magnoliophyta that resemble the other examined eukaryotic taxa (Fig 8C). As discussed above, eukarya can form larger and more complex MP than prokarya within a single domain. In other words, although prokarya use all 20 AA they may not take full advantage of AA diversity. Moreover, when comparing the number of UniRef50 entries for model organisms from each taxa, eukarya utilize more MP than prokarya although *E*. *coli* comes close (Table 1). We hypothesize that, by balancing f_packing_ and f_polar_, a larger overall number of unique MP can be constructed, which benefits eukarya on a functional level and contributed to the rise of f_polar_.

**Table 1:** Number of examined membrane proteins per taxonomic group.

Taxonomic group	Number of examined membrane proteins (UniRef50 entries) for taxonomic group	Model organism representing taxonomic group	Number of examined membrane proteins (UniRef50 entries) for model organism
Bacteria	167,292	–	–
Eukarya	73,363	–	–
Actinobacteria	24,441	*Mycobacterium tuberculosis*	310
Proteobacteria	72,224	*Escherichia coli*	847
Archaea	5,812	*Haloferax volcanii*	106
Magnoliophyta	8,850	*Arabidopsis thaliana*	2,602
Dikarya	10,581	*Saccharomyces cerevisiae*	1,011
Mammalia	13,349	*Homo sapiens*	4,924
Sauropsida	4,503	*Gallus gallus*	944
Actinopterygii	6,194	*Danio rerio*	1,421
Insecta	10,111	*Drosophila melanogaster*	909

The biosynthetically more costly new AA invariably increased AA costs in correlation to f_polar_ (Fig 8D). Nonetheless, mammalia, dikarya, and, most notably, magnoliophyta achieved high E_H_ levels at relatively low costs; the AA cost of magnoliophyta is even at the level of prokaryotes (Fig 8D). This efficiency is achieved by reducing TM helix length as compared to prokaryotes and other eukaryotes (Fig 8E), which further emphasizes Σf and E_H_ as parameters of MP diversity. On the other hand, actinopterygii, sauropsida, and insecta afforded relatively high costs. While magnoliophyta synthesize essentially all AA *de novo*, dikarya, which still have this ability, are already less affected by cost, suggesting that diversified food networks can reduce the effective AA cost. Such networks may have allowed insecta to achieve the highest f_polar_ levels. However, because of the complex lifestyles and multicellular nature of metazoa, it remains ambiguous whether this level reflects a most advanced MP assortment or merely evolutionary history as suggested by their f_polar_ correlation with node number (Fig 8B). In sum, we propose that AA preferences of taxa relate to evolutionary history, overall proteome diversity (functional sophistication), and lifestyle, which determines effective AA cost.

### AA composition of extinct MP

The decline of Ala, Gly, Pro, Arg, Val, and Leu as a function f_Phe_ (Fig 7A and 7B) suggests that those AA were both present when MP protein synthesis first took place and essential to early MP structures. The remaining AA then rose from either low levels because they became more useful to MP over time or from zero as they were incorporated into the genetic code and became available for the first time. For example, the above listed AA lack Ser and Thr that are believed to precede the presence of Arg in the genetic code (Table 2). Ser and Thr may have risen from low levels in relation to functional demands, an altered chemical environment, or advances in inserting MP into the membrane. The early importance of anionic Arg could relate to its ability to anchor proteins to anionic lipids [28, 46] which supports its classification as an anchoring residue (Fig 4E). Subsequently its decline as the principal anchoring residue resulted from competition with Lys and the emergence of the superior Tyr and Trp anchors.

**Table 2:** Predicted amino acid composition of primordial membrane proteins.

AA in order of proposed entry into genetic code^a^	f estimate based on f_Phe_ = -2.24^b^^,^^c^	f estimate based on f_Phe_ = 0^c^
Gly	2.8	2.7
Ala	5.5	4.8
Asp	0.16	0.20
Val	4.7	4.0
Pro	0.94	0.94
Ser	0.12	0.44
Glu	0.05	0.13
Thr	0.75	0.97
Leu	4.9	4.6
Arg	1.2	0.85
Asn	0	0.09
Ile	0	1.1
Gln	0	0.15
His	0	0.02
Lys	0	0.05
Cys	0	0.00
Phe	0	0
Tyr	0	0
Met	0	0
Trp	0	0
SUM	21.1	21.0

^a^according to ref. [38]

^b^assuming the presence of only the first ten AA of the genetic code

^c^calculated using S2 Table

With the f_Phe_ coordinate relating to evolutionary history (Fig 7A), it can be followed back in time to predict the AA composition of the earliest forms of life. We had described f_X_(f_Phe_) using linear functions for f_Phe_≥1.5 (Fig 7A and 7B and S8 Fig). However, to allow slower rates of AA incorporation in earlier evolution (f_Phe_<1.5), we modeled f_X_(f_Phe_) for the wider f_Phe_ range using exponential functions (Fig 7C and S9 Fig). Table 2 summarizes the predicted AA frequencies for this model at time f_Phe_ = -2.24 and when assuming that only the first ten AA were present. Packing, polar and hydrophobic frequencies were estimated at 9.2, 9.6 and 0.9, respectively, implying that structural diversity arose almost entirely from the combinatorial possibilities afforded by packing interactions. The primordial TM helix length was predicted at 21.1 residues (Table 2), which is slightly shorter than their contemporaries (Fig 8E). At time f_Phe_ = 0, the genetic code putatively expanded to 16 AA and packing, polar and hydrophobic frequencies adapted to 8.5, 9.7 and 1.7, respectively (Table 2). That is, polar contributions increased at the expense of packing interactions. Most of the polar increases were predicted to stem from increased Ser and Thr frequencies, which may indicate an elevated demand on MP structural diversity. Among the newly acquired AA, Ile was most successful and Gln and Asn added novel polar interactions. At 21.0 AA, the TM length would essentially be unchanged. The low level of new AA means that entropic considerations favor their usage, which may make their rise a byproduct of nearly neutral net evolution. Nonetheless, the steep rise of e.g. Ser and Ile further suggests that evolutionary pressure directly connects to functional advantages. In this context, we point out similar f values for Trp across taxa (Fig 7B). Trp is both assumed to enter the genetic code last [38] and the most costly AA to synthesize [45]. Nonetheless, in apparent association to (a) distinct functional advantage(s), Trp incorporation was essentially complete by the time the accessible f_Phe_ window was reached (f_Phe_ = 1.5; S8 and S9 Figs). Finally, we note that the decline of packing and rise of polar AA may be "actively" encouraged by CpG hypermutability [47]. In other words, driving organisms to higher AA diversity would be an evolutionary direction that was successful enough to find a biochemical implementation.

## Conclusions

In the absence of all 20 natural AA, biological organisms operated with conceptually simpler and less diverse MP than extant organisms. For most AA, diversification follows predictable relationships between old and new AA (universal ratios) with the position of biological taxa on the f_polar_ coordinate relating to their evolutionary history, proteome diversity, and effective AA cost. As indicated by the sustainability of f trends to higher n values relative to bacteria (Figs 4 and 5), the f_packing_-f_polar_ balances attained by eukarya accomplish a seemingly deeper integration of TM helix bundles (higher level of MP complexity). In other words, more decisive helix-helix interactions per helix appear possible in eukaryotic MP. Further relationships of E_H,_ Σf and AA cost with n and taxa reiterate that achievable MP diversity (functional sophistication) and AA cost are the drivers of MP proteome evolution. Moreover, E_H_ and Σf investments in small, single-domain MP of eukarya and bacteria, respectively (Fig 1), indicate an advantage in maximizing MP diversity for these groups (2≤n≤7 in eukarya). If an MP folds unsuccessfully, it has to be degraded or refolded, which increases effective biosynthetic costs and may have conferred an evolutionary advantage to single-domain MP. However, this circumstance also implies that, if a more efficient folding pathway exists at higher n, transitions to such n can be advantageous overall. It is remarkable that the seemingly old-fashioned packing interactions were retained most by eukarya for 8≤n≤12 (Fig 5). The diminished integration of MP bundles in this bracket may indicate that folding pathways became more difficult at higher n and that packing interactions made them "safer". We therefore propose that foldable MP structures become more limited for n≥8 with the majority of n≥13 MP no longer able to achieve a deeply integrated helix bundle, resulting in the formation of truly independent domains. In sum, the relatively simple analysis of f(n) trends and taxonomic f associations provided fundamentally new insight into MP architecture, composition, and evolution.

## Methods

### Choice of sequence database

As genomes of an increasing number of organisms are sequenced, redundancies arise from closely related sequences. To cover sequence space evenly, clustered databases have been developed [48]. For example, the UniProt Reference Clusters (UniRef) database provides coverage of the sequence space at three resolutions. UniRef100 combines identical sequences and sub-fragments from any organism into a single entry (cluster). A cluster representative is then chosen based on available sequence information [49]. UniRef90 is then built by clustering UniRef100 sequences such that each cluster is composed of sequences with at least 90% sequence identity to and 80% overlap with the seed sequence [49]. UniRef50 is analogously constructed by clustering UniRef90 seed sequences that maintain at least 50% sequence identity to and 80% overlap with the longest sequence in the cluster. Sequences identities that exceed 50% generally exhibit homologous protein structures. As such, UniRef50 is expected to virtually capture all currently existing MP structures. Moreover, on a structural level MP are often homologous at lower sequence similarity, implying that redundancies in protein structure will be present. There are even more stringent databases available [50] but this would fix AA types even at non-conserved sites to the cluster representative. For the purpose of discerning f(n) trends, blurring the difference between conserved and non-conserved sites was not desired, therefore our study employed the UniRef50 database. We note that UniRef50 generally uses the best-annotated database entry as cluster representative, which in rare instances produced entries outside of a selected taxonomic group. For example, 1.4% of entries in the bacterial dataset are from eukarya and the eukaryotic dataset exhibits bacterial entries at 0.4% (Table 1). For individual taxonomic classes (Table 1), such entries increased somewhat (2–8%) when also counting annotation ambiguities as foreign entries. However, even the removal of all such entries merely shifted the original f(n) close to reported uncertainties and to retain even coverage of sequence space f(n) was calculated with all entries present.

### Calculation of AA frequencies (f)

UniRef50 cluster representatives, which exhibited location tags "Multi-pass membrane protein [SL-9909]" or "Single-pass membrane protein [SL-9904]", protein existence codes 1–3, and the desired taxonomic association were initially selected. UniProt classifies protein existence in five categories (http://www.uniprot.org/help/protein_existence) where classes 4 and 5 refer to predicted and uncertain proteins, respectively, which were excluded from analysis. Entries were further required to exhibit "helical" or "discontinuously helical" transmembrane annotations, which resulted in the numbers of examined UniRef50 database entries summarized in Table 1. TM helix border predictions were taken directly from UniProt entries. The frequency, f, of finding an AA, X, in a TM helix is given by the count of X divided by the number of examined helices. The uncertainty in f arising from sampling a finite database size (count of examined TM helices) was estimated to be 2·√(count/4). The uncertainty in f from errors in TM border predictions was estimated using Monte Carlo simulations. For this purpose, the standard deviation between sequence-based TMHMM 2.0 TM border predictions and structure-based estimates contained in UniProt records were calculated for the following proteins. Aquaporin AqpM (PDB: 2f2b, UniProt: AQPM_METTM), heart cytochrome c oxidase (PDB: 5xdq, Uniprot: COX1_BOVIN), free fatty acid receptor 1 (PDB: 4phu, Uniprot: FFAR1_human), DsbB (PDB: 2zuq, Uniprot: DSBB_ECOLI) and facilitated glucose transporter member 3 (PBD: 4zw9, Uniprot: GTR3_HUMAN). Predicted helix borders of all studied UniRef50 records were then varied randomly within a Gaussian distribution of standard deviation 3.8 with the stipulation that the overall helix length cannot shrink by more than five residues. We gratefully acknowledge the use of the GNU Scientific Library, gnuplot and Grace.

### Calculation of AA diversity and biosynthesis cost of TM helix-constituting AA

The Shannon diversity index (H) is defined as–Σ p_x_ ln p_x_ where the sum extends over all 20 AA and p_x_ is the probability of finding residue X in a studied TM helix, i.e., p_x_ = f_x_ / Σf where Σf denotes the sum of f for all AA. We report the Shannon equitability index, E_H_ = H / ln 20, as measure of TM helix AA diversity. The metabolic cost in terms of high-energy phosphate bonds of synthesizing the constituent AA of a TM helix is–Σ f_x_ ATP(X) where ATP denotes the cost of synthesizing AA X *de novo* in *E*. *coli* [45] and the sum extends over all 20 AA.

## Supporting information

S1 Fig Glycine frequencies along the membrane normal. (a-b) f(1,m) profiles of Gly for eukarya and bacteria where m denotes the residue number relative to the predicted TM helix center. Negative and positive m values indicate orientations toward the N- and C-terminus, respectively, and do not relate to extra- and intracellular orienations. In eukarya, the helix-helix association motif GxxxG [13] was directly observed in contrast to bacteria where f(1,m) peaked in the C-terminal helix half and was lowest at the TM helix center. Helices with an even and uneven number of AA are also plotted separately for reference. Fixed UniRef50/Uniprot TM helix borders were used.
(EPS)

S2 Fig Influence of TM helix border definitions on TM helix amino acid frequencies. For each AA, f(n) was calculated using UniProt TM borders plus Monte Carlo simulations (*black*; see Methods), UniProt TM borders (*red*), and UniProt TM borders shrunk by four residues on each side (*green*), respectively. Solid lines show running averages calculated with a window size of two. The n range was limited to ≤19 because of the scarcity of MP for large n (Fig 1A).
(EPS)

S3 Fig Transmembrane amino acid frequencies as a function of n for actinobacteria and archaea. Solid lines depict running f(n) averages calculated with a window size of two. The n range was limited to ≤19 because of the scarcity of MP for large n (Fig 1A).
(EPS)

S4 Fig Transmembrane amino acid frequencies as a function of n for proteobacteria and dikarya. (EPS)

S5 Fig Transmembrane amino acid frequencies as a function of n for magnoliophyta and sauropsida. (EPS)

S6 Fig Transmembrane amino acid frequencies as a function of n for insecta and actinopterygii. (EPS)

S7 Fig Transmembrane amino acid frequencies as a function of n for metazoa and mammalia. (EPS)

S8 Fig Taxa-specific linear correlations between pairwise amino acid frequencies (universal ratios). For AA X, f_Phe_ and f_X_ in the 2≤n≤7 range were averaged for the nine taxonomic groups shown and correlated using f_X_ = a·f_Phe_ + b. Fitted parameters are summarized in S1 Table.
(EPS)

S9 Fig Taxa-specific exponential correlations between pairwise amino acid frequencies (universal ratios). For AA X, f_Phe_ and f_X_ in the 2≤n≤7 range were averaged for the nine taxonomic groups shown and correlated using f_X_ = a·exp(b·f_Phe_) + c. For AA with rising f_X_ c = 0 was assumed. Fitted parameters are summarized in S2 Table.
(EPS)

S1 Table Universal ratios with respect to Phe assuming f_X_ = a·f_Phe_ + b. (PDF)

S2 Table Universal ratios with respect to Phe assuming f_X_ = a·exp(b·f_Phe_) + c. (PDF)
