# Supplementary material for: Universal principles of membrane protein assembly, composition and evolution
Source: PLoS One. 2019 Aug 15;14(8):e0221372. doi: 10.1371/journal.pone.0221372 (PMC6695178; doi:10.1371/journal.pone.0221372)
Supplement: S1 Table — (PDF) [file pone.0221372.s010.pdf]

S1 Table. Universal ratios with respect to Phe assuming $f_X = a \cdot f_{Phe} + b$

X	a	b
A	-2.25	6.68
C	0.76	-1.06
D	0.03	0.20
E	0.12	0.06
F	1	5.89E-13
G	-1.36	4.34
H	0.27	-0.29
I	0.76	0.92
K	0.26	-0.20
L	-0.43	4.76
M	0.28	0.23
N	0.34	-0.21
P	-0.37	1.42
Q	0.13	0.09
R	-0.25	0.92
S	0.78	-0.10
T	0.15	0.93
V	-1.00	4.33
W	-0.09	0.67
Y	0.75	-0.60
