# Supplementary material for: Universal principles of membrane protein assembly, composition and evolution
Source: PLoS One. 2019 Aug 15;14(8):e0221372. doi: 10.1371/journal.pone.0221372 (PMC6695178; doi:10.1371/journal.pone.0221372)
Supplement: S2 Table — (PDF) [file pone.0221372.s011.pdf]

S2 Table. Universal ratios with respect to Phe assuming $f_X = a \cdot \exp(b \cdot f_{Phe}) + c$

X	a	b	c[1]
A	-0.800	0.729	5.635
C	0.004	2.339	0
D	0.202	0.117	0
E	0.127	0.423	0
F	16.126	0.056	-16.036
G	-0.067	1.422	2.799
H	0.018	1.309	0
I	1.136	0.386	0
K	0.050	0.923	0
L	-0.621	0.366	5.186
M	0.360	0.396	0
N	0.085	0.846	0
P	-0.008	1.725	0.944
Q	0.155	0.406	0
R	-0.910	0.193	1.763
S	0.439	0.595	0
T	0.972	0.118	0
V	-0.954	0.514	4.953
W	-0.093	0.428	0.717
Y	0.123	0.982	0

[1]For AA with rising frequencies c=0 was assumed (see Fig. 7b).
